# Macroscale Superlubricity Enabled by Graphene‐Coated Surfaces

**DOI:** 10.1002/advs.201903239

**Published:** 2020-01-19

**Authors:** Zhenyu Zhang, Yuefeng Du, Siling Huang, Fanning Meng, Leilei Chen, Wenxiang Xie, Keke Chang, Chenhui Zhang, Yao Lu, Cheng‐Te Lin, Suzhi Li, Ivan P. Parkin, Dongming Guo

**Affiliations:** ^1^ Key Laboratory for Precision and Non‐Traditional Machining Technology of Ministry of Education Dalian University of Technology Dalian 116024 China; ^2^ Key Laboratory of Marine Materials and Related Technologies Ningbo Institute of Materials Technology and Engineering Chinese Academy of Sciences Ningbo 315201 China; ^3^ State Key Laboratory of Tribology Department of Mechanical Engineering Tsinghua University Beijing 100084 China; ^4^ Department of Chemistry School of Biological and Chemical Sciences Queen Mary University of London London E1 4NS UK; ^5^ State Key Laboratory for Mechanical Behavior of Materials Xi'an Jiaotong University Xi'an 710049 China; ^6^ Materials Chemistry Research Centre Department of Chemistry University College London 20 Gordon Street London WC1H 0AJ UK

**Keywords:** ambient conditions, graphene, macroscale superlubricity, macroscale surfaces, molecular dynamics

## Abstract

Friction and wear remain the primary modes for energy dissipation in moving mechanical components. Superlubricity is highly desirable for energy saving and environmental benefits. Macroscale superlubricity was previously performed under special environments or on curved nanoscale surfaces. Nevertheless, macroscale superlubricity has not yet been demonstrated under ambient conditions on macroscale surfaces, except in humid air produced by purging water vapor into a tribometer chamber. In this study, a tribological system is fabricated using a graphene‐coated plate (GCP), graphene‐coated microsphere (GCS), and graphene‐coated ball (GCB). The friction coefficient of 0.006 is achieved in air under 35 mN at a sliding speed of 0.2 mm s^−1^ for 1200 s in the developed GCB/GCS/GCP system. To the best of the knowledge, for the first time, macroscale superlubricity on macroscale surfaces under ambient conditions is reported. The mechanism of macroscale superlubricity is due to the combination of exfoliated graphene flakes and the swinging and sliding of the GCS, which is demonstrated by the experimental measurements, ab initio, and molecular dynamics simulations. These findings help to bridge macroscale superlubricity to real world applications, potentially dramatically contributing to energy savings and reducing the emission of carbon dioxide to the environment.

## Graphical abstract

A tribological system is developed using a graphene‐coated ball, graphene‐coated microspheres, and a graphene‐coated plate. Macroscale superlubricity is realized using the developed system on macroscale surfaces under ambient conditions. The fundamental mechanisms of macroscale superlubricity are elucidated by ab initio and molecular dynamics simulations.

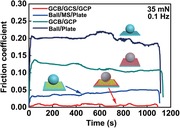

## 1 Introduction

Mechanical friction dissipates about one third to one half of the energy in the world, and around 80% of machine component wear.^1^ Macroscale friction and wear remain the primary energy dissipation in moving mechanical components. It is estimated that nearly one third of fuel is spent to overcome friction in automobiles, while wear greatly reduces the life of mechanical components. Even a modest 20% reduction of friction can substantially contribute to energy savings and reduce carbon dioxide emission.^2^ The removal of friction is known as superlubricity, which is highly desirable for energy saving, environmental benefits and increasing the lifetime of mechanical components.^3^, ^4^ Superlubricity is defined such that the friction coefficient, *µ* is less than 0.01. *µ* is calculated

(1) $$\mu =F_{1}\text{/}F_{n}$$

where the *F_l_* and *F_n_* are lateral and normal forces, respectively. This was originally proposed by Hirano and Shinjo in 1990.^5^ Until very recently, superlubricity has been found only on the nanoscale under ambient conditions.^6^, ^7^ Considerable progress took place in 2012 when microscale superlubricity was observed in graphite in air.^8^ In 2014, macroscale superlubricity was carried out in various atmospheres including dry inert nitrogen (N_2_), argon (Ar), reactive hydrogen (H_2_), and humid air.^9^ Until 2017, microscale superlubricity was demonstrated in ambient air under normal load of 1 µN with a scan size of 1 µm and a scan speed of 2 µm s^−1^.^10^ Lately, macroscale superlubricity has been realized in a nitrogen (N_2_) environment between the diamond‐like carbon and graphene films, through the formation of nanoscrolls of graphene flakes wrapping the nanodiamond particles during the sliding.^2^ It is reported that gas environments have a significant influence on the friction coefficient and wear rate.^11^, ^12^, ^13^, ^14^ In addition, macroscale superlubricity was demonstrated between the inner and outer shells of centimeter‐long double‐walled carbon nanotubes.^1^ Nevertheless, currently macroscale superlubricity has only been demonstrated under special environments or curved nanoscale surfaces. As a result, macroscale superlubricity has not yet been demonstrated on large surfaces under ambient conditions. It was believed that macroscale superlubricity did not exist due to the structural deformation of materials at large scale, except in special environments or curved nanoscale surfaces.^1^, ^15^ Therefore, it is a great challenge to conduct macroscale superlubricity under ambient conditions on macroscale surfaces.

Graphene was found in 2004 with an atomic layer of graphite.^16^ It has extraordinary electronic transport properties, exceptionally thermal conductivity, mechanical stiffness and fracture strength.^16^, ^17^, ^18^, ^19^, ^20^ Graphene is a basic building block for graphitic materials of all other dimensionalities, which can be wrapped up into 0D fullerenes, rolled into 1D nanotubes and stacked into 3D graphite.^21^ Due to the unique physical and mechanical properties, graphene is a promising solid lubricant with an atomically smooth surface and high chemical stability.^22^, ^23^, ^24^ Chemical vapor deposition (CVD) is an effective method to deposit graphene films on insulating materials. Nonetheless, traditional graphene CVD often demands metallic catalysts, such as nickel (Ni) or copper (Cu), and post‐transfer or additional catalyst removal techniques.^10^, ^25^ These complicated processes induce wrinkles, holes, damage and contamination on the as deposited graphene films. It is a big challenge to fabricate a tribological system for macroscale superlubricity with coated graphene on insulating materials.

In this study, macroscale superlubricity was performed under ambient conditions on macroscale surfaces, which was conducted on a newly developed tribological system coated by multilayer graphene (MLG). The MLG were deposited by plasma enhanced CVD (PECVD) powered by radiofrequency at 900 °C on quartz and silica (SiO_2_) surfaces, absent from the catalysts and post‐transfer techniques. PECVD dissociates methane (CH_4_) at 900 °C by radiofrequency power, forming the carbon (C) source of graphene. This does not need the metallic catalysts or conductive substrates as those used in traditional CVD. The fundamental mechanisms of macroscale superlubricity were elucidated by ab initio and molecular dynamics (MD) simulations.

## 2 Results and Discussion

**Figure 1:**
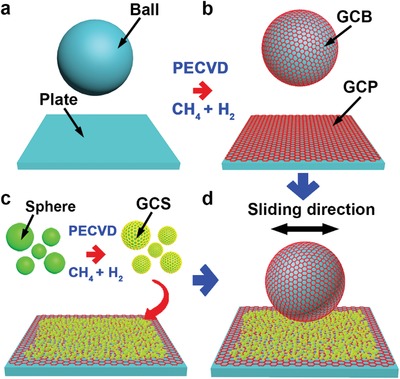
Schematic diagrams of fabrication processes for macroscale superlubricity system from a) a ball and plate b) coated with MLG by PECVD, c) then adding GCS dispersed between the GCB and GCP, and d) finally performing macroscale superlubricity under ambient conditions.

**Figure** **1** illustrates the schematic diagram of the fabrication processes for the macroscale superlubric system under ambient conditions. A ball and plate are coated with MLG by PECVD (Figure 1a,b).^24^, ^25^ PECVD was performed by radiofrequency power at 900 °C, using CH_4_ as the C source and hydrogen (H_2_) as the carrier and protective gas. MLG coated microspheres (GCSs) conducted by PECVD are dispersed between the graphene‐coated ball (GCB) and graphene‐coated plate (GCP) (Figure 1c,d). The microsphere has an average diameter of 8 µm. The GCB is fixed on a developed ball‐on‐plate tribological tester, which is used to perform macroscale superlubricity under ambient conditions (Figure 1d).

**Figure 2:**
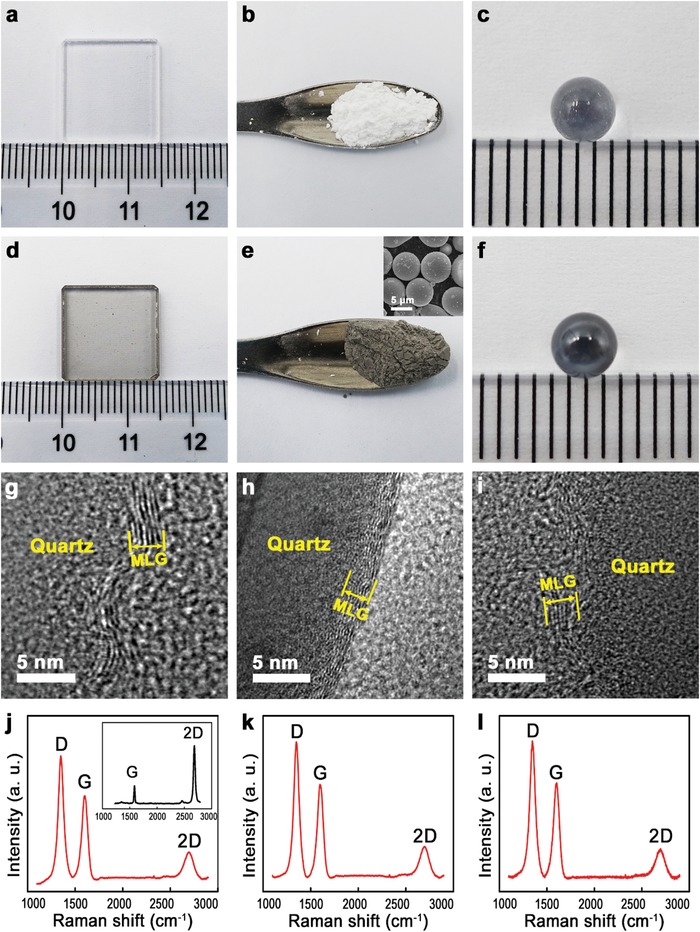
Photographs of pristine a) quartz plate, b) SiO_2_ powder, and c) quartz ball, and after graphene deposition for d) GCP, e) GCS, and f) GCB, g–i) corresponding TEM images, respectively, and j–l) corresponding Raman spectra, respectively. Inset in (e) is the corresponding SEM image. Inset in (j) shows the Raman spectrum of standard high‐quality single layer graphene.

After graphene deposition, all the surfaces turn dark (**Figure** **2**d–f), compared with the bright pristine surfaces (Figure 2a–c). The Ball and plate are made of quartz, and powder or microsphere (MS) are prepared by SiO_2_. GCP, GCS, and GCB were coated with MLG by PECVD simultaneously in a tube furnace, without metal catalysts and post‐transfer treatment, reducing effectively the fabrication processes and contaminations induced by traditional CVD techniques.^10^, ^26^ Eight layers of graphene were coated on the GCP (Figure 2g), GCS (Figure 2h) and GCB (Figure 2i), as observed in transmission electron microscopy (TEM) images. GCS is shown in the scanning electron microscopy (SEM) image in the inset of Figure 2e with diameters at around 8 µm. The surface of GCS is rough, due to the adhered SiO_2_ or dust particles induced during preparation processes by the vendor. Raman spectra of GCP, GCS and GCB are drawn in Figure 2j–l, respectively. Peaks located around 1350, 1580, and 2700 cm^−1^ correspond to D, G, and 2D peaks, respectively.^2^ The D peak is derived from the breathing modes of six‐atom rings and requires a defect to activate. The G peak originates from the E_2g_ phonon at the Brillouin zone center. The 2D peak is the second order of the D peak, which stems from a process where momentum conservation is satisfied by two phonons with opposite wave vectors. It is always present without the requirement of defects for its activation.^27^, ^28^ The ratio between the relative intensity of the G and 2D peaks (*I*_G_/*I*_2D_), indicates a MLG structure on the coated surfaces,^10^, ^29^ which is in good agreement with the TEM results in Figure 2g–i. The relative intensity of the D peak in Figure 2j–l is the maximum, revealing the structural disorder and effects of grain boundaries. Figure 2j–l are typical Raman spectra of defective graphene, which is consistent with those of previous reports.^2^, ^10^ For the high‐quality single layer graphene, defects are basically absent, leading the absence of D peak (Inset of Figure 2j). Due to the single layer of graphene, 2D peak is the highest compared with G peak.^19^ It is reported that the grain size is ≈160 nm in the MLG films, grown by CVD on flat SiO_2_ substrates. Under the same growth processes, the gain size is less than 160 nm on curved and rough surfaces.^10^

**Figure 3:**
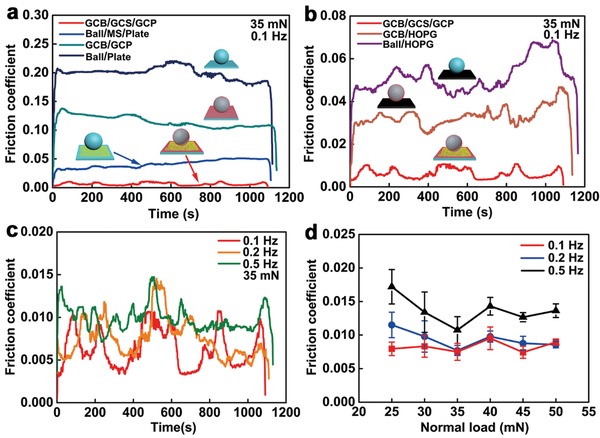
Friction coefficient of a) GCB/GCS/GCP, Ball/MS/Plate, GCB/GCP, Ball/Plate, b) GCB/GCS/GCP, GCB/HOPG, Ball/HOPG as a function of time, and friction coefficient of GCB/GCS/GCP as c) a function of time and d) normal load at different sliding frequencies.

**Figure** **3** pictures the friction coefficient as a function of time under normal load for different tribological systems. Displacement of the cantilever was calibrated and measured by a dual‐frequency laser interferometer with the nonlinearity error less than 4.2 nm (10705A, 5517C, Keysight Technologies, USA). Lateral force was measured by the double‐leaf cantilevers. After calibration, the cantilevers had a lateral spring constant of 1000 N m^−1^. The resolution and accuracy of the developed tribometer was 0.01 mN, and the maximum load was 1 N. In Figure 3a, the average friction coefficients of Ball/Plate, GCB/GCP, Ball/MS/Plate, and GCB/GCS/GCP are 0.2, 0.11, 0.04, and 0.006 respectively under a normal load of 35 mN at a sliding frequency of 0.1 Hz. Macroscale superlubricity is realized by the GCB/GCS/GCP tribological system under ambient conditions. For a comparison, highly oriented pyrolytic graphite (HOPG) (Nanjing XFNANO Materials Tech Co., Ltd., China) was used as the tribo‐pair with ball and GCB, as presented in Figure 3b. The average friction coefficients of Ball/HOPG, GCB/HOPG, and GCB/GCS/GCP are 0.05, 0.03, and 0.006, respectively under 35 mN at 0.1 Hz. At a sliding time of 1200 s, the average friction coefficient of GCB/GCS/GCP under 35 mN at 0.1, 0.2, and 0.5 Hz is 0.006, 0.008, and 0.01 (Figure 3c), respectively, corresponding to the sliding speeds of 0.2, 0.4, and 1 mm s^−1^. The dynamic videos of macroscale superlubricity under 35 mN at 0.1 and 0.5 Hz are displayed in Movies S1 and S2 in the Supporting Information, respectively. In Figure 3d, macroscale superlubricity is realized under normal loads from 25 to 50 mN, at a sliding speed of 0.2 mm s^−1^ for 1200 s under ambient conditions on macroscale surfaces. To identify the function of the MS, the friction coefficient of GCP/MS/GCP is shown in Figure S1 in the Supporting Information. Under 30 mN at 0.1 Hz, the average friction coefficient of GCP/MS/GCP is 0.017, and the friction coefficient decreases first from 0.04 to 0.016 under 50 mN at 0.1 Hz, then increasing sharply to 0.7. The experiments demonstrate that the MLG on curved and flat surfaces plays the decisive role for the robust macroscale superlubricity under ambient conditions.

**Figure 4:**
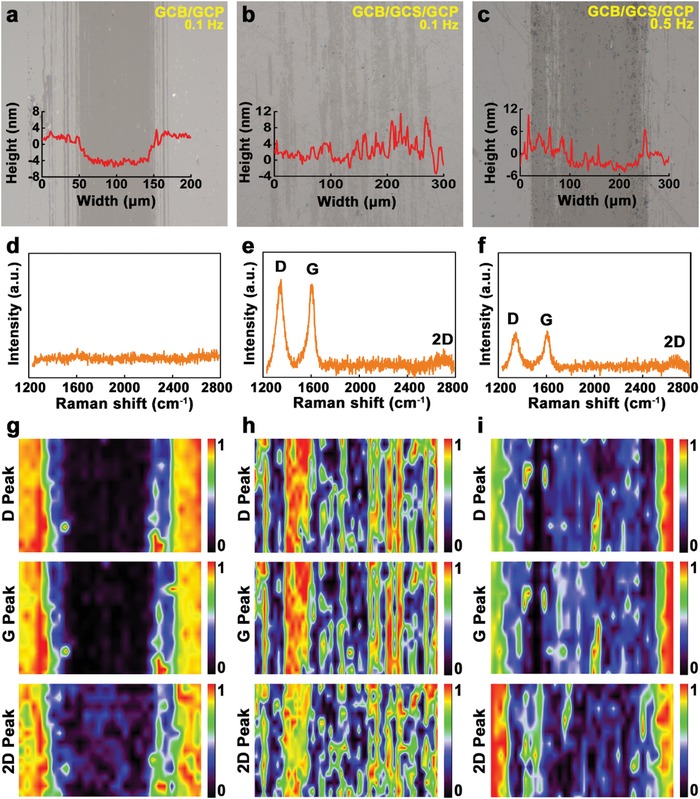
Optical images of the wear track of the GCP after friction tests for a) GCB/GCP (measured at 0.1 Hz), b) GCB/GCS/GCP (measured at 0.1 Hz), and c) GCB/GCS/GCP (measured at 0.5 Hz), d–f) corresponding Raman spectra, respectively, and g–i) corresponding Raman mapping, respectively.

**Figure** **4** illustrates the optical images of the wear track of the GCP after friction tests for a) GCB/GCP (measured at 0.1 Hz), b) GCB/GCS/GCP (measured at 0.1 Hz), and c) GCB/GCS/GCP (measured at 0.5 Hz), corresponding Raman spectra d–f), respectively, and g–i) corresponding Raman mapping (, respectively. The wear width and depth of GCB/GCP are about 100 µm and 8 nm (Figure 4a), respectively. In Figure 4a, there are small scratches that are absent in Figure 4b,c, which might be induced by the rough surface of the pristine ball (Figure S1c in the Supporting Information). Adding the GCS as shown in Figure 4b,c, the small scratches disappear due to the diameter of GCS at around 8 µm. There is no signature of graphene on the wear track of GCB/GCP (Figure 4d–g). This is consistent with the friction coefficient of 0.11 in Figure 3a. In Figure 4b, the width is ≈280 µm, and it is difficult to identify the depth of wear. The width and depth in Figure 4c are about 230 µm and 5 nm, respectively. The higher position in Figure 4b,c, might be attributed to the accumulation of exfoliated flakes of graphene during sliding. Adding GCS dramatically reduces the wear of the GCP, and graphene is found after sliding, which is confirmed by Raman spectra and mapping (Figure 4e–i). The wear resistance of GCB/GCS/GCP at 0.1 Hz is better than that at 0.5 Hz, which is confirmed by their Raman spectra (Figure 4e,f) and mapping (Figure 4h,i). The relative intensities of D peaks in Figure 4e,f decrease, and the 2D peaks exhibit amorphous characteristics after sliding, compared to those in Figure 2j prior to sliding. This means that the defects of MLG in the wear track are improved. The decrease of intensity on D peaks is different from previous reports, in which the intensity of D peaks usually increases after sliding.^30^, ^31^ In this study, eight layers of graphene, i.e., MLG were deposited by PECVD. However, in previous work, single layer graphene was deposited by traditional CVD,^30^ and two or three layers of graphene were deposited through evaporation of ethanol, resulting in the coverage area of graphene at less than 25% on the substrate.^31^ In traditional CVD, only one layer graphene was deposited, which is easy to be worn out during sliding, increasing the defects of graphene, as well as the intensity of D peak. Through the evaporation of ethanol, two or three layers were deposited, while the coverage area was less than 25%. The exfoliated flakes of graphene formed debris during sliding, which is difficult to cover the bare area left over 75%. The transferred film generated by debris induced new defects of graphene, leading to the increase of intensity of D peak. However, in this study, there are eight graphene layers deposited by PECVD, inducing the transferred films on the wear track made by debris derived from the exfoliated flakes during sliding. This improves the defects of graphene, resulting in the decrease of intensity for D peak. Debris is observed on wear tracks in Figures S4 and S5 in the Supporting Information. Without the GCS, debris are pushed at the edge of the wear track in Figures S2a,d in the Supporting Information. With the GCS, debris is found on the wear tracks in Figure S2b,e,c,f in the Supporting Information. The wear track of GCB/GCS/GCP at 0.1 Hz (Figure S2b, Supporting Information) is the slightest among the three tracks, and GCS is embedded in the wear track. The height of wear debris on the wear track of GCB/GCS/GCP at 0.1 Hz reaches 1.32 µm (Figure S3b, Supporting Information). Therefore, debris contributes immensely to the realization of macroscale superlubricity.

**Figure 5:**
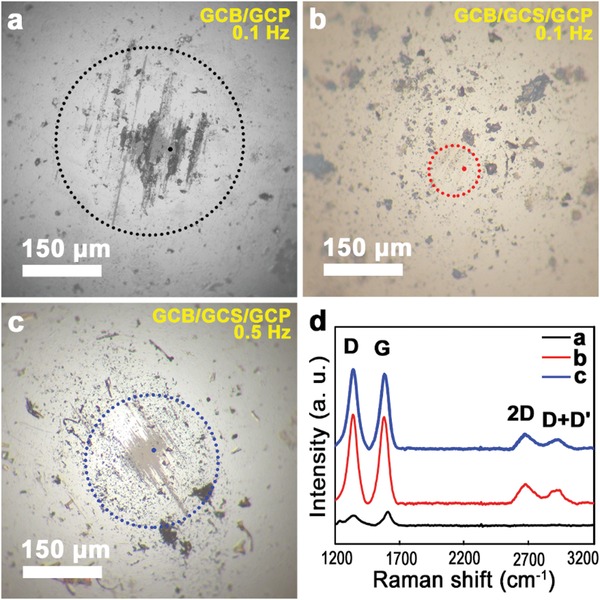
Optical images of the wear areas on the GCB after friction tests under 35 mN for a) GCB/GCP (measured at 0.1 Hz), b) GCB/GCS/GCP (measured at 0.1 Hz), and c) GCB/GCS/GCP (measured at 0.5 Hz), and d) their Raman spectra taken from the corresponding small dots. Wear areas are marked by dotted circles in each figure.

**Figure** **5** shows the optical images of the wear areas on the GCB after friction tests under 35 mN for GCB/GCP (measured at 0.1 Hz), GCB/GCS/GCP (measured at 0.1 Hz), and GCB/GCS/GCP (measured at 0.5 Hz), and their Raman spectra. In Figure 5a, the graphene was worn out. The wear diameter is 131 µm, the wear depth is 1.1 µm, and the wear volume is 7230.7 µm^3^. Wear rate, *W*_s_ is calculated^12^

(2) $$W_{s}=\;\;\frac{V}{NS}$$

where *V*, *N*, and *S* are the wear volume, normal load and sliding distance, respectively. The wear rate calculated is 4.3 × 10^−4^ mm^3^ N^−1^ m^−1^. It is difficult to identify the wear area without the marking shown with a red dotted circle in Figure 5b, demonstrating the enormous reduction of wear for materials under macroscale superlubricity. In Figure 5b, the diameter of the grey color is 91 µm. If this was the wear diameter, and the wear depth was 0.5 µm. This would lead to the worn out of graphene, due to the coated graphene with thickness <3 nm in Figure 2g–i. This means the coated graphene has no wear with adding the GCS, i.e., the GCB has no wear in macroscale superlubricity. In Figure 5d, the 2D peak is absent without the GCS taken from the black dot in Figure 5a, meaning the worn out of graphene. With the GCS, D peaks decrease, and D + D′ peaks appear at around 2940 cm^−1^ in Figure 5d. D + D′ peak is the combination of phonons with different momenta, requiring a defect for its activation.^27^ In Figure 5b,c, the graphene is present, the D peak decreases, and D + D′ peak appears compared with the pristine Raman spectra in Figure 2l. Due to a transfer film of exfoliated graphene flakes, D peak decreases and new defects are generated, activating the presence of D + D′ peak. During the sliding, a few layers of graphene at the top of GCB were worn out, leading to the generation of defects in the wear area and activating the D + D′ peaks. To investigate the wear conditions of the GCS, the SEM images prior to and after sliding under 35 mN at 0.5 Hz are depicted in Figure S4 in the Supporting Information. The GCS has no observable wear after sliding (Figure S4b, Supporting Information). Exfoliated graphene flakes were transferred on the GCS (Figure S4b,c, Supporting Information), which is beneficial for the macroscale superlubricity.

**Figure 6:**
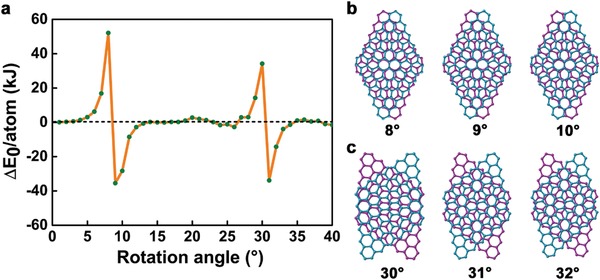
a) Variation of energy per atom as a function of rotation angle, and snapshots of the supercell rotated with the top layer of graphene at rotation angles of b) 8–10°, and c) 30–32°.

To elucidate the origin of macroscale superlubricity, ab initio calculations were performed, as illustrated in **Figure** **6**. The rotation angles of the supercell from 8 to 10° and 30–32° are depicted in Figure 6b,c, respectively. The graphene is a symmetrical honeycomb structure, and therefore 30° is a period for rotation angles. In this regard, the rotation angle calculated is from 0 to 40°. The variation of energy per atom, ∆*E*_0_ per atom increases monotonically from 0 to 8° (Figure 6a). When the rotation angle reaches 8°, the ∆*E*_0_ per atom is 52.1 kJ, reaching the maximum value during the rotation from 0 to 40°. However, the ∆*E*_0_ per atom is −35.4 kJ, when the rotation angle arrives at 9°, meaning the decreasing of ∆*E*_0_ per atom from 8 to 13°. This results in the spontaneous rotation from 8 to 13° for the top layer graphene, unnecessary for the input of external energy and force. It is in good agreement with previous reports on microscale and nanoscale superlubricity for incommensurate contact of graphene and graphite.^32^, ^33^ For the rotation of graphene, 30° is a period, and hence spontaneous rotation happens from 30 to 34° with the ∆*E*_0_ per atom values varying from positive to negative.

**Figure 7:**
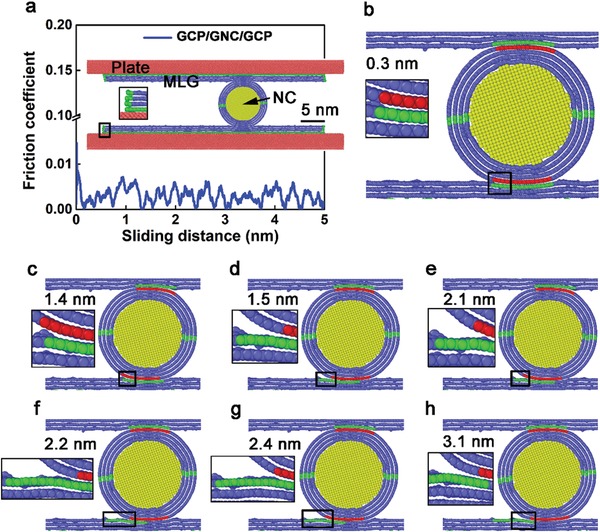
a) Friction coefficient as a function of sliding distance, and snapshots of atomic configurations of the top plate wrapped with the graphene sliding at b) 0.3, c) 1.4, d) 1.5, e) 2.1, f) 2.2, g) 2.4, and h) 3.1 nm. Inset in (a) shows the side view of the constructed MD model. Insets marked by the bigger black squares show the corresponding enlarged images marked by the smaller black squares in each figure.

To illuminate the mechanisms of macroscale superlubricity, MD simulations are depicted in **Figure** **7**. The friction coefficient of the GCP/GNC/GCP is 0.003, which is consistent with the experimental results of macroscale superlubricity (Figure 3). GNC represents MLG coated nanocylinder (NC). In the inset of Figure 7a, the green color denotes the fixed C atoms of graphene, blue color means the graphene, yellow color represents the silicon (Si) atoms of the NC, and red color signifies the Si atoms of plates. In Figure 7b–h, contact atoms between the GCP and GNC are marked by green and red colors respectively, to recognize the movement of GNC during the sliding. The dynamic sliding video is displayed in Movie S3 in the Supporting Information. During the sliding of the top GCP, the upper contact atoms of GNC adhere on the sliding atoms, without basically any movement. However, the lower contact atoms of the GNC slide backward for 1 atom relative to the lower GCP at a distance of 1.4 nm (Figure 7c), and then slide forward quickly for 5 atoms at a distance of 1.5 nm (Figure 7d). At a distance of 2.1 nm (Figure 7e), the lower contact atoms of GNC slide backward for 1 atom, and then slide forward for 5 atoms at 2.2 nm (Figure 7f). At 2.4 nm (Figure 7g), the lower contact atoms of GNC slide backward for 1 atom, and then slide forward for 6 atoms at 3.1 nm (Figure 7h). From Movie S3 in the Supporting Information and Figure 7, the GNC waggles and slides to facilitates the superlubricity, which is different from the previous findings which showed rolling, sliding and transfer of exfoliated materials.^34^ Nevertheless, the mechanism suggested in this study agrees well with the ab initio simulations in Figure 6. The rotation of graphene between 8–13° and 30–34° happens spontaneously, due to the reduction of energy. This contributes to the swinging and sliding of the GNC, due to the incommensurate contact between two layers of graphene during sliding. The error bars for the friction coefficient in Figure 3d are attributed to the asperity contact surface and spontaneous rotation, between 8–13° and 30–34° in a rotation period for the incommensurability contact during sliding.

**Figure 8:**
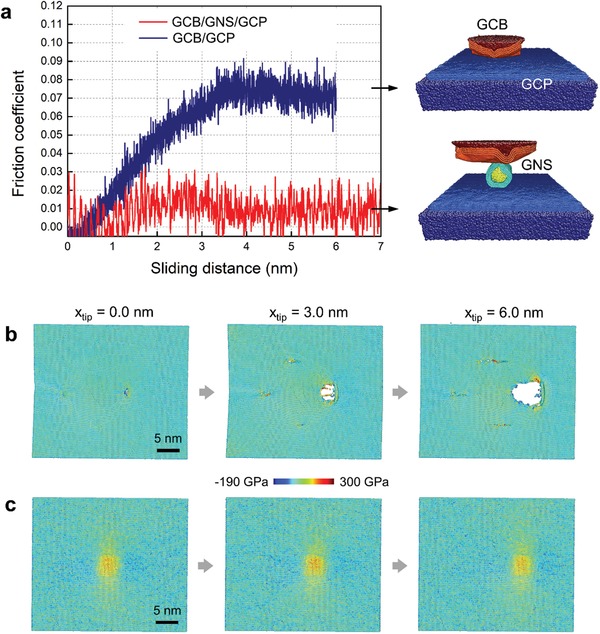
a) Friction coefficient of the GCB/GCP and GCB/GNS/GCP as a function of sliding distance, and typical atomic configurations at different distance of the topmost graphene sheet attached on plate b) without and c) with GNS. a) Dark red, orange, cyan, yellow, light blue and dark blue refer to the scratching tip, MLG on the tip, MLG wrapped on NS, NS, MLG on the plate and plate, respectively. b,c) The colors are coded according to the hydrostatic stress, and atoms with positive and negative values correspond to the tensile and compressive stresses, respectively.

**Figure** **8** shows the friction coefficient of the GCB/GCP and GCB/GNS/GCP as a function of sliding distance and typical atomic configurations at different sliding distances with and without a MLG coated nanosphere (GNS). During scratching, the normal load was applied on the tip for 1500 nN, scratching speed was 0.3 m s^−1^ along the length direction of the plate, and scratching distance was 6 and 7 nm for the GCB/GCP and GCB/GNS/GCP systems, respectively. Without GNS, the friction coefficient increases linearly when the sliding distance is less than 3.5 nm (Figure 8a). After 3.5 nm, the friction coefficient reaches a saturated value around ∼0.8, which is close to 0.11 measured in experiments (Figure 3a). After adding the GNS, the friction coefficient decreases dramatically. When *x*_tip_ < 3.5 nm, the friction coefficient slightly increases to a peak value of about 0.015, and then decreases to 0.01. After *x*_tip_ > 3.5 nm, the friction coefficient stabilizes at ≈0.01, approaching the value of macroscale superlubricity in experiments (Figure 3).^35^ The variation of friction coefficient is attributed to the relatively high normal load of 1500 nN and high sliding speeds. The hydrostatic stress state of the topmost graphene sheet attached on the plate is shown in Figure 8b at different sliding distances. The dynamic sliding without GNS of the topmost graphene sheet is displayed in Movie S4 in the Supporting Information, and their typical atomic configurations are shown in Figure S5a in the Supporting Information. Prior to scratching, the topmost graphene sheet is ruptured under the maximum compressive stress of −190 GPa, when exerted to the normal load of 1500 nN (Figure 8b). When scratching to 3.0 and 6.0 nm, the tensile stress attains the maximum of 300 GPa, breaking the C—C bonds and rupturing the topmost graphene sheet (Figure 8b). After scratching, a wear track is left behind the scratching tip. Through adding GNS, the hydrostatic stress is greatly decreased, without either rupture or formation of a wear track during sliding, and the wear resistance of the topmost graphene sheet is remarkably improved (Figure 8c). The dynamic sliding process and typical atomic configurations of the topmost graphene sheet with GNS, is displayed and shown in Movie S5 in the Supporting Information and Figure S5b in the Supporting Information, respectively.

To analyze the mechanism of superlubricity with GNS, a front view of Movie S5 in the Supporting Information is displayed in Movie S6 in the Supporting Information to observe clearly the movement of the GNS. From Movies S5 and S6 in the Supporting Information, the mechanism of superlubricity with GNS is seen to be due to swinging and sliding, which is consistent with the MD simulated results with different models in Figure 7. The adding of GNS contributes two aspects towards the superlubricity: one is the buffer function, and the other is stress dispersion through swinging. GNS is responsible for the buffer regime between the scratching tip and graphene sheet. The addition of GNS reduces the contact area that slides against the topmost graphene sheet.^2^ The swinging of GNS disperses the contact point continuously, avoiding the concentration of stress on the topmost graphene sheet.

Lateral force, *F_l_* of microscale superlubricity in graphite is presented^36^

(3) $$F_{1}=\;\;2\gamma_{g}L$$

where γ_g_ is the surface energy of the graphite basal plane, and *L* is the contact width. The calculated results using Equation (3) is in good agreement with experimental results previously reported.^37^, ^38^ The average number of MS is 54.4 on the contact areas, which was measured from the SEM images on 78 contact areas after sliding of macroscale superlubricity, as depicted in Figure S6a in the Supporting Information. The number of MS is selected as 55 on contact area, and the average normal force, *F_n_* on each MS is 0.636 mN. γ_g_ is 0.227 J m^−2^.^39^ From Hertz contact model, the contact radius, *r* is addressed between the MS and a rigid surface^40^

(4) $$r\;\;=\;\;{\left(3RF_{n}\text{/}4E*\right)}^{1/3}$$

where *R* is the radius of the MS, and E* is the effective elastic modulus. E* is described^40^

(5) $$E*=\;\;{\left[\left(1\;\;-\;\;v_{\text{s}}^{2}\right)\text{/}E_{s}+\;\;\left(1\;\;-\;\;v_{\text{p}}^{2}\right)\text{/}E_{p}\right]}^{-1}$$

where *E* is the elastic modulus, and *v* is the Poisson's ratio of the MS (s) and the plate (p). The elastic modulus and Poisson's ratio are 73.3 GPa^41^ and 0.17,^42^ respectively. The *r* calculated is 331, 351, 370, 387, 402, and 417 nm under normal loads of 25, 30, 35, 40, 45, and 50 mN, respectively. This confirms that the adding of MLGCMS could reduce the contact area. The maximum calculated aspect contact pressures are 1.32, 1.41, 1.48, 1.55, 1.61, and 1.66 GPa for a GCS under loads of 25, 30, 35, 40, 45, and 50 mN, respectively. These pressures are higher than 1 GPa calculated in microscale superlubricity.^10^ It is reported that when the contact area exceeds a critical value, the superlubricity is destroyed.^5^ From SEM images, 639 scratching widths were measured on the contact areas, and the average scratching width is 3.45 µm (Figure S6b–d, Supporting Information). The scratching width is induced by several MSs and exfoliated flakes of graphene for a lot of sliding. According to Equations (1) and (3), the friction coefficient of macroscale superlubricity calculated is 0.0025, which is consistent with the simulated and experimental results.

## 3 Conclusion

In summary, the GCB/GCS/GCP tribological system was fabricated simultaneously in a tube furnace by PECVD, in which there are no metal catalysts and post‐transfer process. Macroscale superlubricity was realized on a flat quartz plate coated by MLG under ambient conditions, in which the normal load varies from 25 to 50 mN at a sliding speed of 0.2 mm s^−1^ for 1200 s. After sliding, the defects on the wear areas of GCB and wear tracks of GCP are improved by adding GMS, as confirmed by Raman spectra. The wear on GCB and GCP in the macroscale superlubricity system is the lowest, which is difficult to discern compared with other systems under the same sliding conditions. Ab initio and MD simulations were employed to elucidate the mechanism of superlubricity, according to the experimental conditions observed for macroscale superlubricity. It was found that the swinging and sliding of GCS plays a crucial role for the realization of superlubricity. Our results pave a way for the design and fabrication of high‐performance devices with macroscale superlubricity, as well as for energy savings and reduction of emissions to the environment.

## 4 Experimental Section

Quartz plates had a length of 15 mm, a width of 15 mm, and a thickness of 2 mm. Prior to deposition, all the quartz plates were machined in a polisher (UNIPOL‐1200S, Shenyang Kejing Auto‐instrument Co., Ltd., China) by lapping, mechanical polishing (MP), and chemical mechanical polishing (CMP) sequentially. During lapping and polishing, the polishing pressure and rotation speed were 40.6 kPa and 80 rpm, respectively. The machining time was 3, 10, and 25 min for lapping, MP and CMP, respectively. Abrasive papers with a mesh size of 3000, polyurethane, and nubuck were used as the lapping, MP and CMP pads, respectively. Deionized water was the lapping solution. MP slurry consisted of 2 wt% alumina, 0.1 wt% sorbitol, and deionized water. CMP slurry was made from the MP slurry through adding citric acid to a pH value of 5.4. The developed machining processes and slurries for the quartz plates were efficient and environment‐friendly, respectively. Surface roughness *R*_a_, root mean square (rms), and peak‐to‐valley (PV) values were 0.87 ± 0.03, 1.09 ± 0.04, and 9.13 ± 0.65 respectively on the polished surface, as illustrated in Figure S9a in the Supporting Information. After deposition with MLG, the surface roughness was improved for *R*_a_, rms and PV to 0.76 ± 0.04, 0.96 ± 0.05, and 8.90 ± 0.59, correspondingly (Figure S7b, Supporting Information). The error bars for surface roughness were due to the five measurements on the polished surfaces prior to and after growth of graphene.

The balls (Donghai County Zhenke Quartz Product of Co., Ltd., China) and SiO_2_ powder or MS (Hunyuan County Fuhong Mineral Product of Co., Ltd., China) were made of quartz, and the diameter of balls was 4 mm. Balls and polished plates were ultrasonically cleaned in alcohol for 10 min, and then dried by compressed air. Balls, plates and powder were put in a tube furnace of PECVD (Anhui BEQ Equipment Technology Co., Ltd., China) simultaneously to deposit graphene films. Prior to deposition, the tube furnace was evacuated for 8 min to a vacuum of 5 Pa. Then, the tube furnace was heated, and the temperature was increased to 900 °C within 50 min with a flux of H_2_ at 20 standard cubic centimeter per minute (sccm). When the tube furnace was at 900 °C, the temperature was kept constant for 10 min with a flux of H_2_ at 20 sccm. During the deposition of graphene films, the power of the plasma was 250 W, and the flux of CH_4_ and H_2_ was 16 and 20 sccm, respectively. The deposition time was 30 min. To ensure the uniformity of graphene on the SiO_2_ powders, the temperature and pressure of CH_4_ and H_2_ were kept constant at 900 °C, 16 and 20 sccm during the deposition, which was significant for the uniformity of deposited graphene. In addition, prior to deposition, the SiO_2_ powders were ultrasonically dispersed in alcohol and sprayed on the quartz plate. The dispersed SiO_2_ powders were heated at 60 °C for 5 min, and then they were put in the tube furnace of PECVD for deposition. After deposition, the PECVD system was turned off, and the temperature was allowed to naturally reduce to room temperature. Prior to and after deposition, photographs of balls, plates and powder were taken by a camera (DSC‐RX100, SONY, Japan). Wear tracks and coated MLG were characterized and measured by a field emission SEM (Verios G4 UC, FEI, USA), confocal Raman microscope (inVia Reflex, Renishaw, UK), TEM (Tecnai F20, FEI, USA) and laser scanning confocal microscope (LSM 700, ZEISS, Germany). TEM samples of the MLGCB were prepared by focused ion beam technique (Helios G4 CX, FEI, USA). MLGCMS were ultrasonically dispersed in alcohol for 1 min, and then picked up by a Cu grid used for preparation of TEM specimens. TEM samples of the GCP were prepared manually. First, two GCP specimens were glued face‐to‐face, cut by an ultrasonic cutter to a disc of 3 mm in diameter (Gatan 601, USA), lapped by a disc grinder system (Gatan 623, USA), polished by a waterproof abrasive paper with a mesh size of 2000, thinned at the central area by a dimple grinder to a thickness of ranging from 10 to 30 µm (Gatan 656, USA), and finally thinned by a precision ion polishing system (Gatan 691, USA). Surface roughness was measured by a non‐contact surface profilometer (NewView, 5022, ZYGO, USA).

Tribological tests were performed on a developed homemade tribometer.^22^ The tribometer had a reciprocating ball‐on‐plate configuration. It was driven by a bending actuator with high displacement (PL140, PI, Germany). The normal load was applied by a precision high‐load linear stage (M‐414, PI, Germany) and measured by a precision force sensor. Frictional force sensor was designed and measured by the double‐leaf cantilevers. Lateral force was measured by the double‐leaf cantilevers. The cantilevers were calibrated by stiffness according to the method used for those in atomic force microscopy (AFM). During the tribological tests, the normal load varied from 25 to 50 mN, sliding length was 2 mm, and sliding frequencies changed from 0.1 to 0.5 Hz. For each test, the experiments were repeated for five times, and the average value was used for friction coefficient.

Ab initio calculations were carried out in the Vienna ab initio simulation package (VASP) using the projector augmented wave (PAW) method.^43^, ^44^ All energies were calculated by Perdew–Burke–Ernzerhof exchange‐correlation potentials, based on generalized gradient approximation of density functional theory.^45^, ^46^, ^47^ A supercell consisting of 64 C atoms was used to perform ab initio calculations. The supercell included two‐layer graphene, and each layer contained 32 C atoms. Prior to calculation, the supercell was relaxed to optimize the total energy and force. The length of C—C bonds was 1.42 Å, and the interlayer distance was 24 Å. The criterion of energy convergence was set as 10^−5^ eV, and a k‐point grid of 21 × 21 × 1 was employed to calculate the electronic structure. The cutoff energy of a plane wave was 500 eV. During the calculation of energy, the top layer of graphene was rotated for 40° from the initial equilibrium position, and the atoms were migrated stepwise for 40 steps until reaching the final positions.

The GCP/GNC/GCP tribological performance was conducted by MD simulations. Both plate and NC were set as rigid bodies composed of a silicon single crystal. The two plates and NC were wrapped with four‐layer graphene to perform the superlubricity analysis, which was consistent with the macroscale superlubricity from the experiments. One‐layer graphene was fixed on the plate to simulate the coated MLG on the quartz plate during the experiments. Two‐layer atoms were fixed on two edges of the four‐layer graphene wrapped on two plates. For the four‐layer graphene wrapped on the NC, two‐layer atoms were fixed along the horizontal diameter. Each plate had a length of 44 nm, a width of 6.4 nm, and a thickness of 2 nm. The diameter of the NC was 6 nm. The four‐layer graphene on the plate had a length of 40 nm and a width of 6.4 nm. As the length of graphene was over six times that of the diameter of the NC, the boundary effect was effectively suppressed. The four‐layer graphene had a thickness of 1.035 nm, and the interlayer distance was 0.3445 nm. The interactions of C—C, Si—Si, and C—Si covalent bonds were simulated by the adaptive intermolecular reactive empirical bond order (AIREBO), Tersoff and 6–12 Lennard‐Jones (LJ) potentials, respectively.^23^, ^48^ For the LJ potential, the total energy U was calculated

(6) $$U(r)\;\;=\;\;4\varepsilon \left\{{\left(\sigma \text{/}r\right)}^{12}-\;\;{\left(\sigma \text{/}r\right)}^{6}\right\}$$

where ε is the depth of the potential well, σ is the distance in which the interparticle potential is zero, and *r* is the distance between pairs atoms.^49^ Herein, ε_C‐Si_, σ_C‐Si_, and *r*_C‐Si_ were 0.0096 eV, 3.0 Å, and 10 Å, respectively.^23^ The simulations were carried out at 300 K using the Nosé‐Hoover thermostat and LAMMPS code. The atomic configurations were displayed by the OVITO software. Prior to calculations, the GCP and GNC were separately and integrally relaxed for 200 ps, respectively. During the simulations, the top GCP was applied for 500 nN and moved downward at 10 m s^−1^. When touching the GNC, the downward movement of GCP was stopped. During sliding, the normal load was applied and consistently kept at 500 nN on the top GCP, NC and bottom GCP. Sliding was performed on the length direction of the GCP, and the speed and distance were 5 m s^−1^ and 5 nm, respectively.

To investigate the tribological properties with and without GNS, MD models were constructed on GCB/GCP and GCB/GNS/GCP_._ The Ball, NS and plate were made of amorphous SiO_2_,^50^, ^51^, ^52^ and all of them were wrapped by four‐layer graphene. The ball and NS had diameters of 20 and 5 nm, respectively. The plate had a length of 36.4 nm, a width of 31.7 nm, and a thickness of 6.5 nm. The bottom atoms of the plate were fixed for a thickness of 0.5 nm. Four‐layer graphene were adhered on the plate with a length of 33.9 nm and a width of 29 nm. Two edges of graphene sheet along the length direction of the plate were fixed, to constrain the overall translational movement during sliding. The ball was truncated as the scratching tip, and rough contact surface was created to trap the NS stably beneath the tip under stress. The dynamic trapping process of GNS was displayed in Movie S7 in the Supporting Information. The atomic interactions in graphene and amorphous SiO_2_ were calculated by AIREBO and Tersoff potentials, respectively.^23^, ^48^ A typical 6–12 LJ potential was employed to describe the van der Waals adhesive interaction between the MLG and ball, MLG and NS, and between graphene layers. The LJ parameters were set ε = 0.023 eV, σ = 0.30 nm for graphene‐SiO_2_ interaction, and ε = 0.0024 eV, σ = 0.34 nm for graphene layers. The scratching simulations of GCB/GCP and GCB/GNS/GCP systems were performed with LAMMPS code, and visualized by AtomEye.^23^ The simulations were conducted at 300 K using a Nosé‐Hoover thermostat.

## Conflict of Interest

The authors declare no conflict of interest.

## Author Contributions

Z.Y.Z. and Y.F.D. contributed equally to this work. Z.Y.Z. and D.M.G. conceived the projects. Z.Y.Z. wrote the paper. All authors commented on the manuscript prior to submission. Y.F.D. designed and performed the experiments of macroscale superlubricity. S.L.H. and S.Z.L. conducted the molecular dynamics simulations. L.L.C. and K.K.C. carried out the ab initio simulations. W.X.X. carried out the chemical mechanical polishing experiments. Z.Y.Z., Y.F.D., F.N.M., C.C.Z., Y.L., C.‐T.L., and I.P.P. analyzed the mechanisms of macroscale superlubricity. All authors discussed the results and commented on the manuscript.

## Supporting information

Supporting Information

Supplemental Movie 1

Supplemental Movie 2

Supplemental Movie 3

Supplemental Movie 4

Supplemental Movie 5

Supplemental Movie 6

Supplemental Movie 7
