# Supplementary material for: Macroscale Superlubricity Enabled by Graphene‐Coated Surfaces
Source: Adv Sci (Weinh). 2020 Jan 19;7(4):1903239. doi: 10.1002/advs.201903239 (PMC7029642; doi:10.1002/advs.201903239)
Supplement: Supplementary file 1 — Supporting Information [file ADVS-7-1903239-s001.pdf]

Copyright WILEY-VCH Verlag GmbH & Co. KGaA, 69469 Weinheim, Germany, 2020.

ADVANCED SCIENCE
Open Access

# Supporting Information

for *Adv. Sci.,* DOI: 10.1002/advs.201903239

# Macroscale Superlubricity Enabled by Graphene-Coated Surfaces

*Zhenyu Zhang,* Yuefeng Du, Siling Huang, Fanning Meng, Leilei Chen, Wenxiang Xie, Keke Chang, Chenhui Zhang, Yao Lu, Cheng-Te Lin, Suzhi Li,* Ivan P. Parkin, and Dongming Guo*

Supporting Information for

# Macroscale superlubricity induced by graphene coated surfaces

Zhenyu Zhang,†* Yuefeng Du,† Siling Huang, Fanning Meng, Leilei Chen, Wenxiang Xie, Keke Chang, Chenhui Zhang, Yao Lu, Cheng-Te Lin, Suzhi Li,* Ivan P. Parkin, Dongming Guo

Prof. Z. Zhang, Y. Du, S. Huang, F. Meng, L. Chen, W. Xie, Prof. D. Guo

Key Laboratory for Precision and Non-Traditional Machining Technology of Ministry of Education, Dalian University of Technology, Dalian 116024, China.

E-mail: zzy@dlut.edu.cn

Prof. K. Chang

Key Laboratory of Marine Materials and Related Technologies, Ningbo Institute of Materials Technology and Engineering, Chinese Academy of Sciences, Ningbo 315201, China.

Prof. C. Zhang, Prof. C. Lin

State Key Laboratory of Tribology, Department of Mechanical Engineering, Tsinghua University, Beijing 100084, China.

Dr. Y. Lu

Department of Chemistry, School of Biological and Chemical Sciences, Queen Mary University of London, London E1 4NS, UK.

Prof. S. Li

State Key Laboratory for Mechanical Behavior of Materials, Xi'an Jiaotong University, Xi'an 710049, China.

E-mail: lisuzhi@xjtu.edu.cn

Prof. I. Parkin

Materials Chemistry Research Centre, Department of Chemistry, University College London, 20 Gordon Street, London, WC1H 0AJ, UK.

†These authors contributed equally to this work.

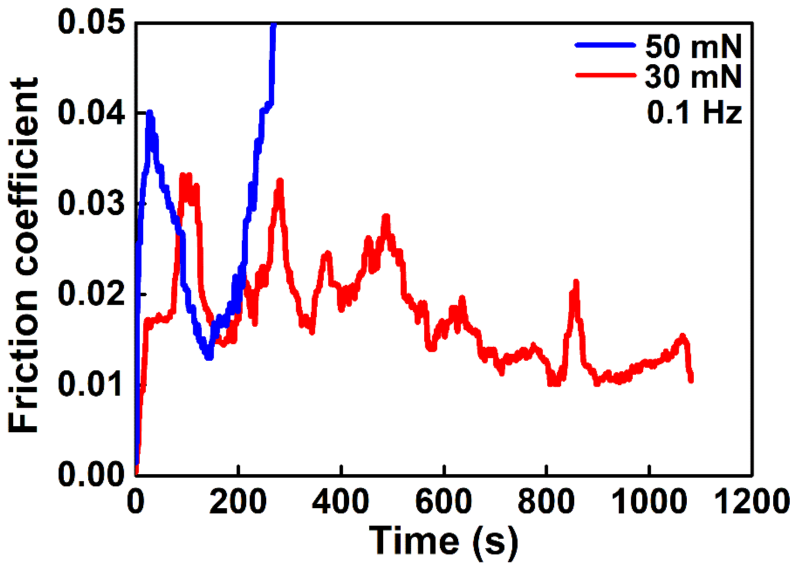


Figure S1 Friction coefficient of GCB/MS/GCP as a function of time under 30 and 50 mN.

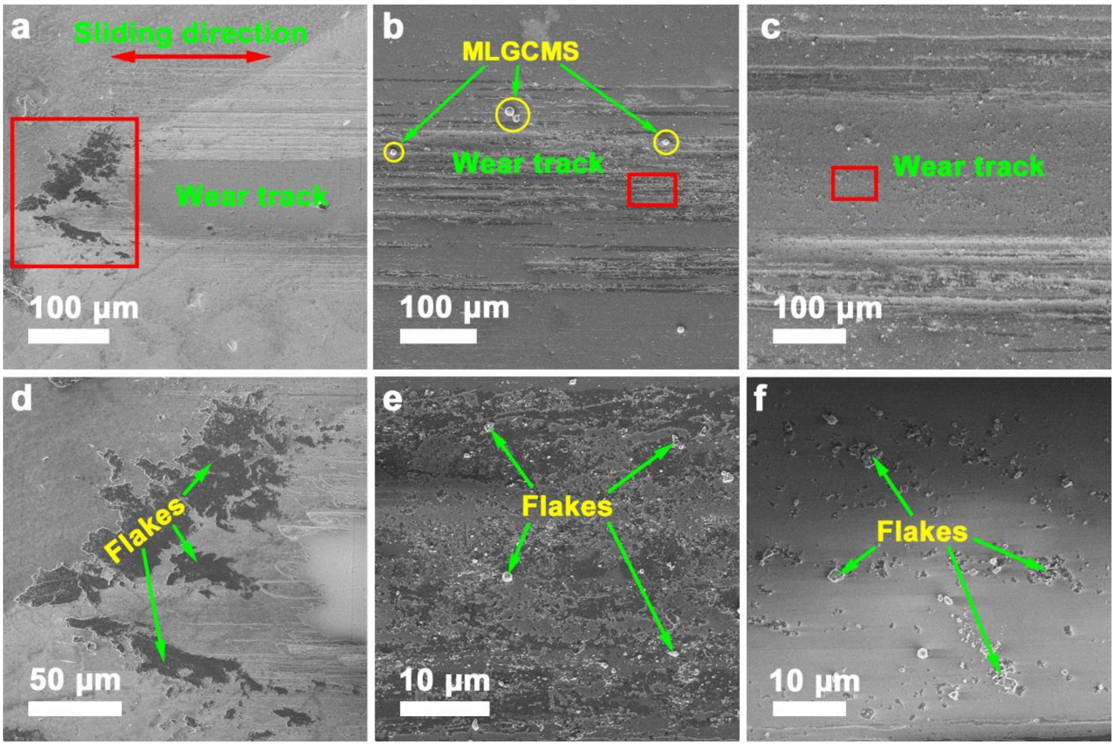


Figure S2 SEM images of wear tracks under 35 mN for (a), (d) GCB/GCP at 0.1 Hz, (b), (e) GCB/GCS/GCP at 0.1 Hz, and (c), (f) GCB/GCS/GCP at 0.5 Hz at low (a), (b), (c) and high (d), (e), (f) magnifications taken from the corresponding areas marked by red squares.

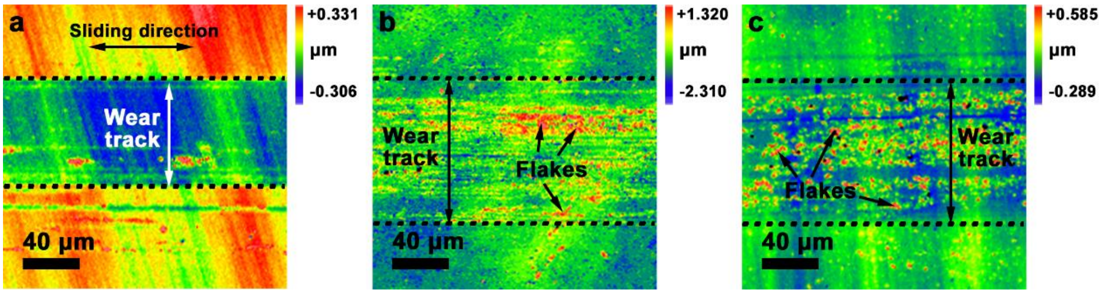


Figure S3 Surface morphology and roughness on the wear tracks under 35 mN for (a) GCB/GCP at 0.1 Hz, (b) GCB/GCS/GCP at 0.1 Hz, and (c) GCB/GCS/GCP at 0.5 Hz.

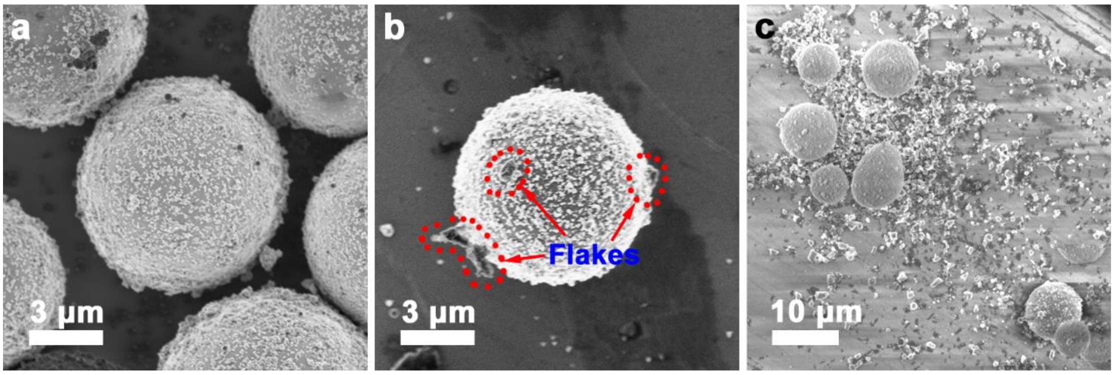


Figure S4 SEM images of the GCS prior to (a) and after (b), (c) sliding under 35 mN for GCB/GCS/GCP at 0.5 Hz.

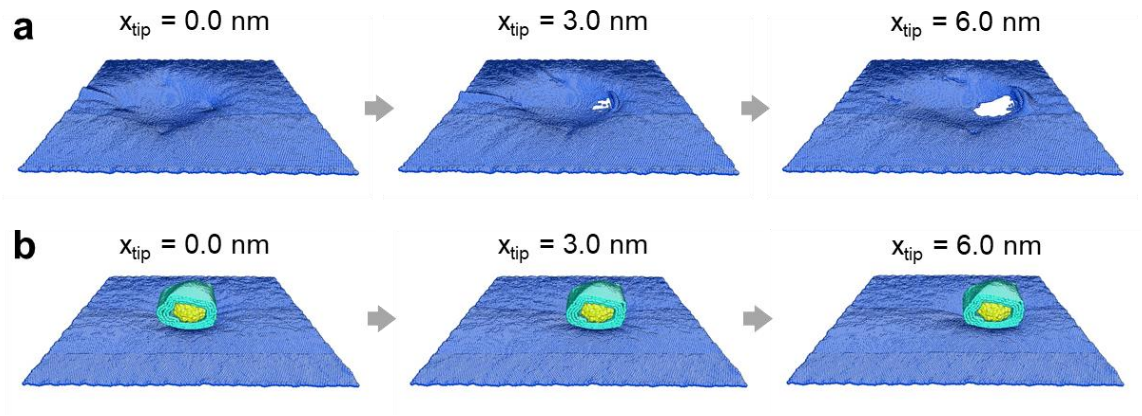


Figure S5 Typical atomic configurations of the topmost graphene attached on the plate at different sliding distance in the (a) GCB/GCP and (b) GCB/GNS/GCP systems.

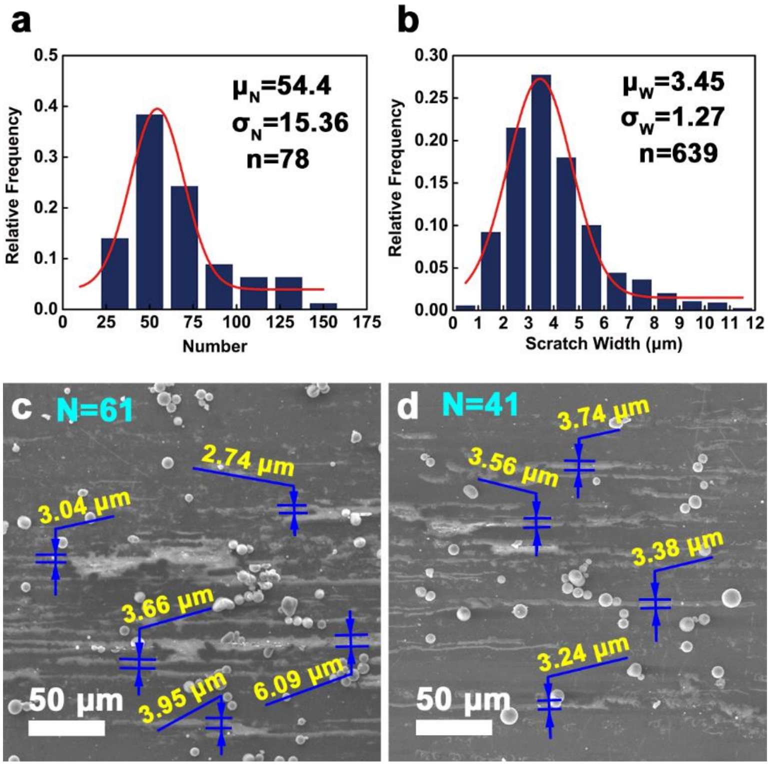


Figure S6 Distribution of frequencies on (a) number of MS at contact areas and (b) scratching widths induced by GCS, and their typical (c), (d) SEM images. Red curves in (a) and (b) are fitted Gaussian distributions.

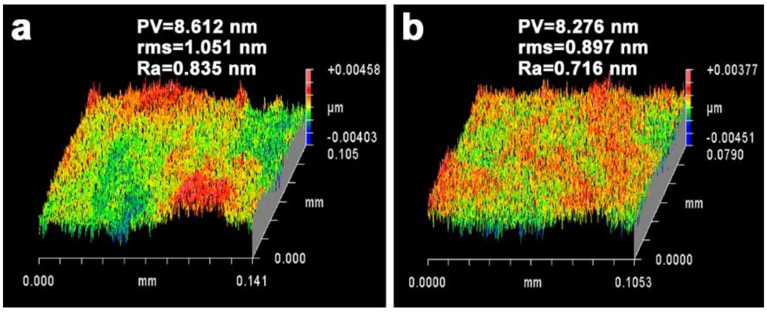


Figure S7 Surface roughness on (a) the polished surface of quartz plate and (b) after deposition of MLG.
